# Analysis of the Effect of Parameters on Fracture Toughness of Hemp Fiber Reinforced Hybrid Composites Using the ANOVA Method

**DOI:** 10.3390/polym13173013

**Published:** 2021-09-06

**Authors:** H. K. Madhusudhana, M. Prasanna Kumar, Arun Y. Patil, R. Keshavamurthy, T. M. Yunus Khan, Irfan Anjum Badruddin, Sarfaraz Kamangar

**Affiliations:** 1 School of Mechanical Engineering, KLE Technological University, Hubballi 580031, India; 2 Department of Mechanical Engineering, University BDT College of Engineering, Davangere 577004, India; drmpkumar.dvg@gmail.com; 3 Department of Mechanical Engineering, Dayananda Sagar College of Engineering, Bangalore 560078, India; keshavamurthy.r@gmail.com; 4 Department of Mechanical Engineering, College of Engineering, King Khalid University, Abha 61421, Saudi Arabia; yunus.tatagar@gmail.com (T.M.Y.K.); magami.irfan@gmail.com (I.A.B.); sarfaraz.kamangar@gmail.com (S.K.)

**Keywords:** natural fiber, hemp fiber, Taguchi method, orthogonal array, ANOVA, fracture toughness

## Abstract

In today’s world, global warming has become a concern. To overcome this, we need to reduce the carbon footprints caused by the production of materials. Much of the time, this is equivalent to the same amount of CO_2_ emissions per tonne of production. This is a serious concern and needs to be overcome by identifying alternative materials to have as minimal a carbon footprint as possible. In this context, hemp fiber is by far the best natural fiber when compared to its peers. As per the survey conducted by the Nova institute, hemp has CO_2_ emissions of only 360 Kg/tonne, whereas jute has CO_2_ emissions of 550 Kg/tonne, kenaf 420 Kg/tonne, and flax 350 Kg/tonne. This paper presents an experimental study of the fracture toughness of hemp-reinforced hybrid composites (HRHC). The effect of the parameters on the fracture toughness behavior of HRHC is studied using the Taguchi technique. It uses different filler combinations with hemp fiber and epoxy. Hemp fiber is used as the reinforcement, epoxy resin is used as a matrix, and banana fiber, coconut shell powder, and sawdust are used as fillers. The experimental plan is prepared using an orthogonal array and analyzed using Minitab software. The obtained results were analyzed using ANOVA and main effects plots. It was observed that the fracture toughness increases with a decrease in thickness. The fracture toughness is affected by the fiber content in the range of 25%–35% and is also affected by the filler materials.

## 1. Introduction

Natural fiber composites have created a lot of interest in place of synthetic fibers because of their low density, vast availability, low cost, and biodegradability. Natural fibers are gaining prominence as a viable alternative to synthetic fibers. This may be seen in the amount of study being done to locate the appropriate material to use for reinforcing instead of synthetic fibers [1,2,3,4,5,6]. As the natural fibers have high stiffness and strength, when they are added to the polymer matrix, the tensile properties of the composites are significantly improved. Flax is one of the most promising natural plant fibers for polymer composites because of its unique features. In natural fiber-reinforced composites, using continuous unidirectional and ideally structured reinforcements is critical for maximizing load-carrying capacity [4,7,8,9,10,11]. Numerous studies have been carried out on the mechanical properties of natural fibers, which are combined with different thermoplastics such as PLA and PHA and thermosets such as phenol-formaldehyde, epoxy, and polyester. These studies have revealed that the bonding between the matrix and the fiber plays a prominent role in the overall mechanical properties of the resulting biocomposite, as the transfer of stress between the fiber and the matrix determines the efficiency of the reinforcement. Composites reinforced with natural fibers are used in different applications in the aerospace, automobile, building, and packaging industries [12,13]. Steel reinforcements have been replaced by carbon- and glass fiber-reinforced composites, as fracture properties are one of the essential requirements of structural members, but the need for environmentally friendly materials has been increasing recently [12,13,14]. Composites reinforced with natural fibers can be considered as better options for structural members if they show enormous strength when subjected to fracture loading. The interface bonding between the matrix and fiber parts in the biocomposite lead to it having good fracture toughness [15,16].

The fracture toughness of biocomposites is forecasted to play an essential role in the future due to there being an increasing demand for it in different applications. Epoxy-based composites reinforced with natural fibers are generally susceptible to brittle fracture when they are subjected to mechanical loading. The tip of a crack in a material appears as a line passing from one location to another. The stress concentration is high at the crack tip. Thus, to obtain displacement and stress field, a crack tip analysis is beneficial. The problem is simplified by converting the two variables into a single variable which is known as the stress intensity factor (K_IC_). The fracture toughness values are helpful in performance evaluation, material characterization, and quality assurance for engineering structures such as oil and gas pipelines, nuclear pressure vessels and piping, petrochemical structures, aircraft, ships, and automotive structures [1,17,18,19]. The application of fracture mechanics concepts to polymers and composites is very much in the primitive stage when compared with metals.

The experimental design can be easily carried out using the Taguchi method, and hence it is very effective. The Taguchi method helps analyze the input function, which is the statistical data, to produce an optimal result. This method has been developed to design experiments to study how the variance and mean of a process are affected by different parameters. Analysis of variance (ANOVA) can be performed on the data obtained from the Taguchi design to determine new values of parameters so that the performance characteristics can be optimized [19,20,21]. The current work has a unique approach to the problem by handling the validation of work with the help of the Design of Experiments. The variation in the outcome is well within the acceptable limits. The novelty of the work is to exhaustively study the fracture toughness behavior of the hybrid composite for a suitable kind of application. However, it is essential to attain an optimal combination of parameters for applications such as automotive interior parts, packaging material for consumer products, etc.

## 2. Materials and Methods

### 2.1. Materials

**Figure 1:**
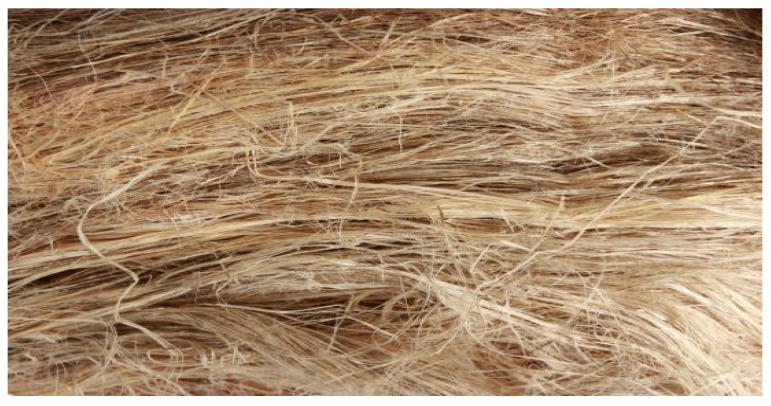
Hemp fiber.

Hemp is used in a wide variety of applications. It is renewable, environmentally friendly, and inexpensive. The hemp fiber (Figure 1) consists of 76% cellulose, 1.5% pectin, 22.4% hemicellulose, and 5.7% lignin. Hemp fiber has properties similar to natural bast fiber and has excellent absorbency, durability, fiber length, strength, and anti-microbial properties. Blending it with other fibers can increase the strength of the fiber. Hemp fiber exhibits a tensile strength of 550–900 MPa, density 1.4 g/m^3^, elastic modulus of 70 GPa, and specific strength of 392.85–642.85 kPa m^3^/kg. It has an elongation of 1.6% and a diameter of 25–600 µm [22]. The details of material suppliers are liste in Table 1.

Banana fiber contains cellulose, hemicelluloses, and lignin. Banana fiber is widely appreciated for its characteristics such as high strength, strong moisture absorption, good luster, light weight, fast moisture absorption and release, slight elongation, easy degradation, and many more. Banana fibers exhibit tensile strengths ranging from 176 MPa to 525 MPa. Banana fibers consist of 15–16% lignin, 14–17% hemicellulose, and 31–35% cellulose. They have an elongation of 1.0–3.5% and a diameter of 80–250 µm.

Coconut is a major plantation crop in Sri Lanka, and coconut millers strive to add value to coconut shells generated as agricultural waste material. Leading coconut millers grinds the shells into a powder to be used by industry as filler or in any other way that would add value. Coconut shell is a waste material that results from some agricultural activities. The density of coconut shell is 1.60 g/cm^3^, and it has high strength and shell particles of sizes between 200–800 μm.

Woodworking operations such as drilling, milling, sawing, planning, etc., produce a byproduct known as wood dust or sawdust. Sawdust consists of fine wood particles. During woodworking, the wood chips and dust are formed at the surface of the work. Sawdust has the following properties: moisture content 10.8, porosity 84%, and density of 210 g/cm^3^.

Epoxy resins are usually brown in color and have a wide range of valuable properties. They can be quickly and easily cured and have low shrinkage during curing. The other mechanical properties include good chemical resistance, high adhesiveness, and high insulation. Epoxies are often used as sealants, adhesives, paints, varnishes, and laminating resins—the properties of epoxy are as follows in Table 2.

### 2.2. Fabrication of Hybrid-Composite

**Figure 2:**
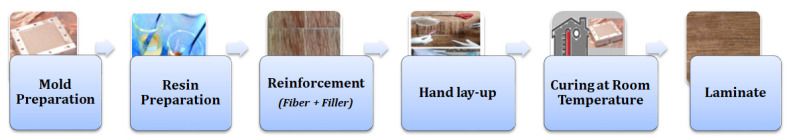
Hand lay-up process.

Hand lay-up is one of the most basic and traditional open molding methods used in composite fabrication. This process requires only the primary resources, and preparation is also simple, as shown in Figure 2. The hemp and banana fibers are soaked in the alkaline solution 5% NaOH at 25 °C for one hour. Then, the fibers are dried in the sunlight till the moisture content is evaporated. The dried fibers are cut to specific lengths and a mat of 300 × 300 mm^2^ is prepared.

### 2.3. Methodology

**Figure 3:**
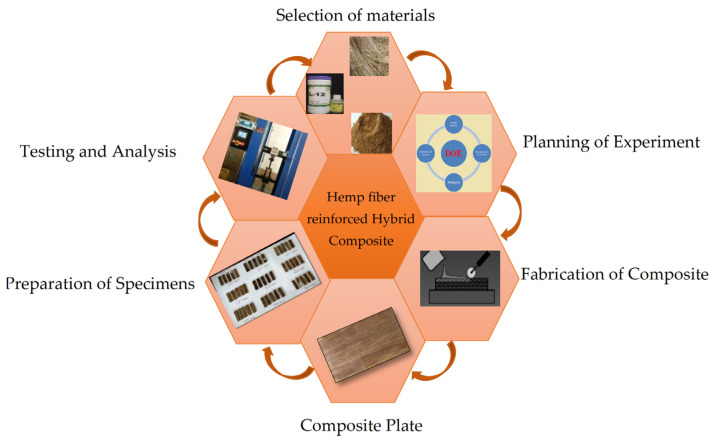
Flow chart of process.

The methodology of the present research work is explained in Figure 3. Material selection is made based on the literature survey. Planning of the experiment is done by using DOE technique, and hand layup is used for the fabrication of specimens.

## 3. Experiments 

**Figure 4:**
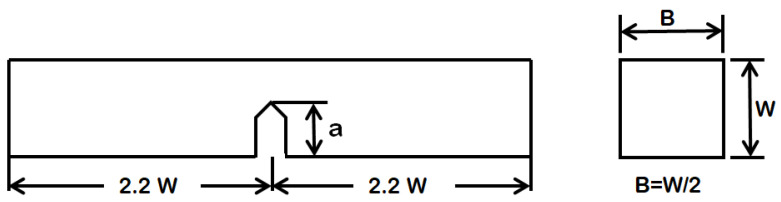
Dimensional view of Single-Edge Notched Bending (SENB) test specimen, Length = 88 mm, Width = 20 mm, Breadth = 10 mm, a/w = 0.45–0.55 mm.

**Figure 5:**
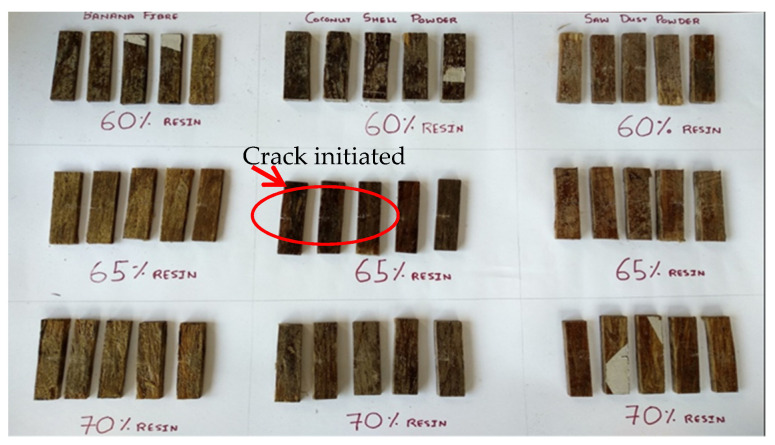
SENB test specimens.

The experiment is carried out by using the hand-layup method. Initially, the chemical treatment is carried out to remove the excess moisture content and improve mechanical properties such as strength, toughness, Young’s modulus, etc. The composite is prepared by reinforcing the different filler materials with hemp fiber using epoxy resin. The specimens are cut according to ASTM D5045 [1], as shown in Figure 4. The Single-Edge Notched Bending (SENB) test specimen is a rectangular specimen with a single edge notch used for fracture test. The fabricated composite laminate and fracture test specimens are shown in Figure 5. 

The planning of the experiment is carried out according to the L9 orthogonal array, as illustrated in Table 3. According to the L9 orthogonal array, input parameters selected are resin content in %, filler material content in %, and thickness in mm. The three levels with three factors are selected for the experiment. Table 4 illustrates the process parameters and the levels for the experiment.

## 4. Results and Discussion

Once the testing is completed, the experimental results are obtained. These results are further analyzed using signal to noise ratio of the Taguchi method and regression analysis. The analysis is carried out using Minitab software (Minitab, LLC is a privately owned company headquartered in State College, State College, PA, USA). Fracture toughness (K_IC_) is the ability of a material to resist fracture against crack propagation. Specifically, fracture toughness testing characterizes resistance to fracture in a neutral environment with a sharp crack. K_IC_ is depicted as the critical value of the stress intensity factor at a crack tip given to produce a catastrophic failure under simple uniaxial loading. 

The fracture toughness is calculated using the formula, and tabulated in Table 5.

(1) $$K_{IC}=\frac{P_{Q}}{(B\left(W{)}^{\frac{1}{2}}\right)}\;f\left(x\right)$$

where, *K_IC_* = Fracture toughness in MPa.√m, *P_Q_* = Ultimate load in kN, *B* = Specimen thickness in mm, *W* = Specimen width in mm, *a* = Crack length in mm, *x* = *a/W*.

The signal to noise ratio analysis is carried out for the fracture toughness values using the Minitab Software and the main effects plots and regression equation are obtained.

The signal-to-noise ratios (S/N ratios) are obtained for the fracture toughness values using Minitab software. The analysis of variance (ANOVA) is carried out for the S/N ratio. From the ANOVA Table 6, it can be concluded that the probability value (*p*-value) for resin content (0.314) is greater than 0.05. Therefore, it is statistically insignificant. The *p*-value for thickness and for the fiber content is less than 0.05. Hence, these two parameters are statistically significant. 

The response table for signal-to-noise ratio is obtained from the Minitab software using a larger is better type, as shown in Table 7. Rank 1 is obtained for fiber content as there is a variation in the mean values. Rank 2 is obtained for thickness, as there is less variation in the mean values. As there is little to no variation in the mean values of resin content, Rank 3 is obtained for it.

**Figure 6:**
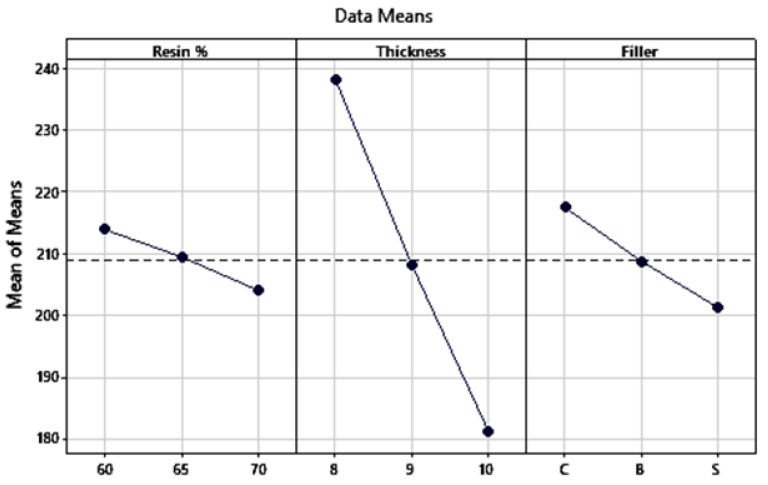
Main effects plot for fracture toughness.

From the main effects plot as shown in Figure 6, it can be observed that maximum fracture toughness is obtained for the combination of 8 mm thickness, 60% resin content, and coconut shell powder as filler.

### 4.1. Fracture Toughness-3 Point Bending Test (Load v/s Displacement Graphs)

The load vs. displacement graphs are plotted for each of the five crack lengths and all nine compositions, as shown in Figure 7, Figure 8, Figure 9, Figure 10, Figure 11, Figure 12, Figure 13, Figure 14, Figure 15.

**Figure 7:**
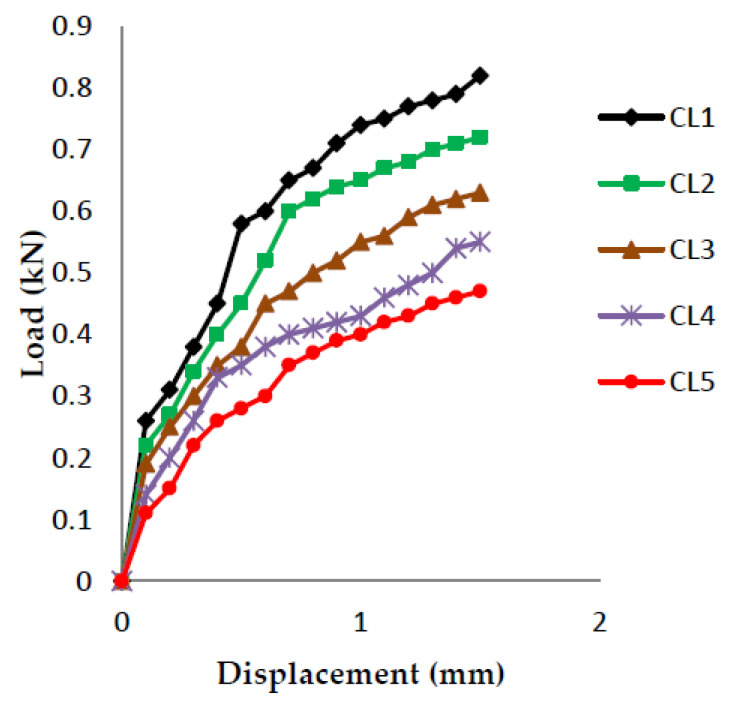
Load vs. Displacement for 60% resin content with banana as filler.

**Figure 8:**
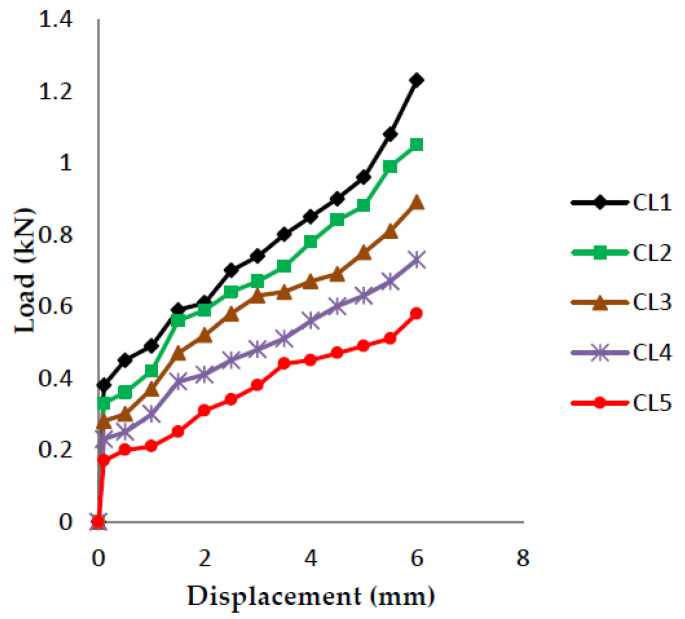
Load vs. Displacement for 60% resin content with coconut shell powder as filler.

**Figure 9:**
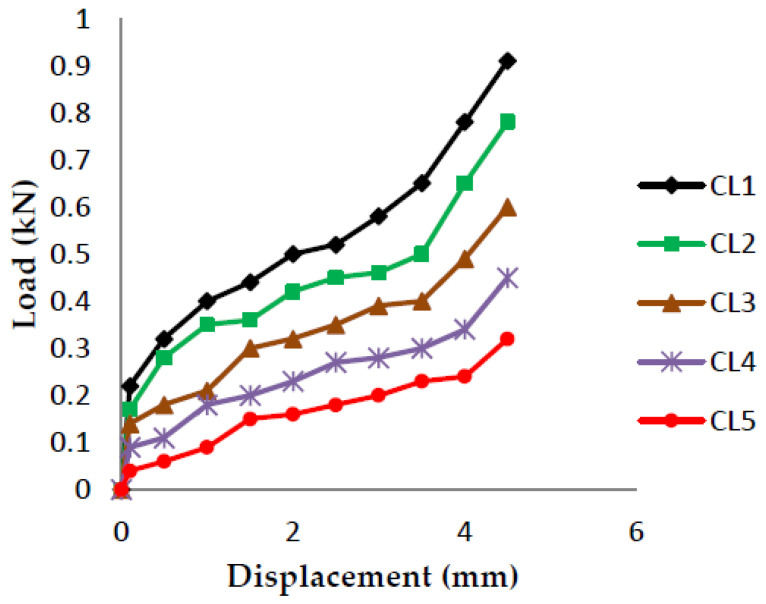
Load vs. Displacement for 60% resin content with sawdust as filler.

**Figure 10:**
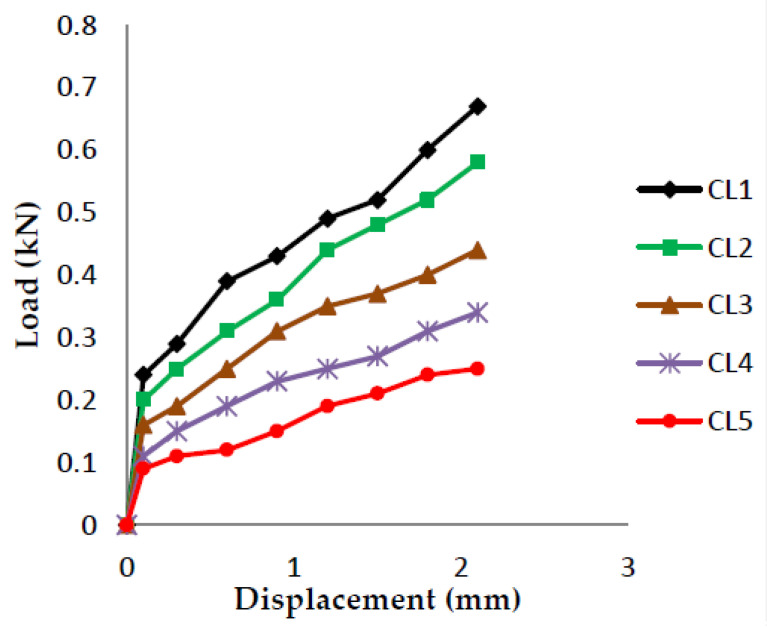
Load vs. Displacement for 65% resin content with banana as filler.

**Figure 11:**
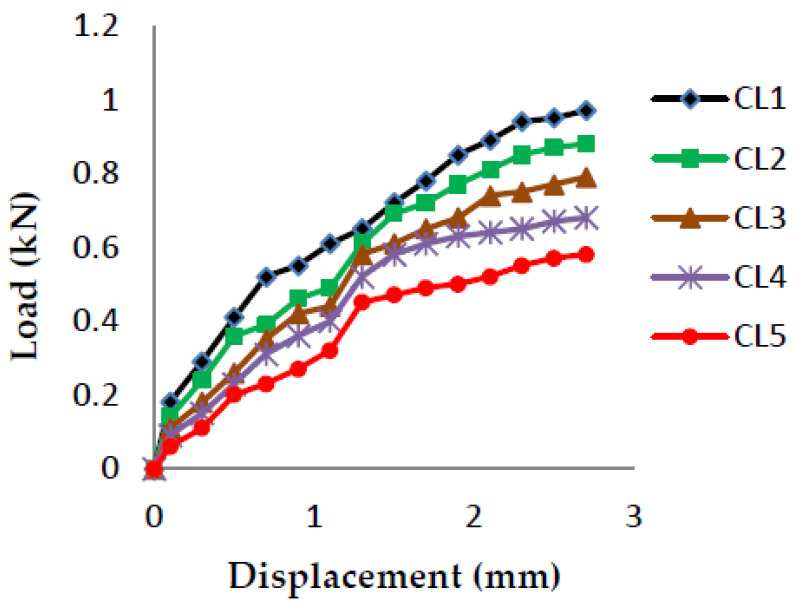
Load vs. Displacement for 65% resin content with coconut shell powder as filler.

**Figure 12:**
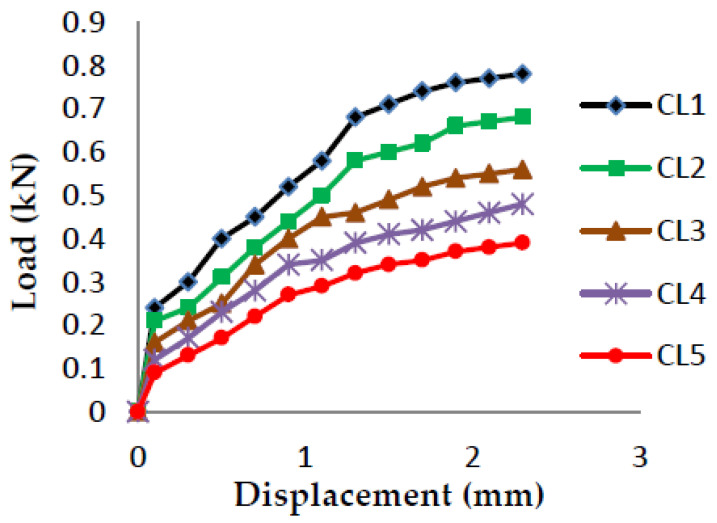
Load vs. Displacement for 65% resin content with sawdust as filler.

**Figure 13:**
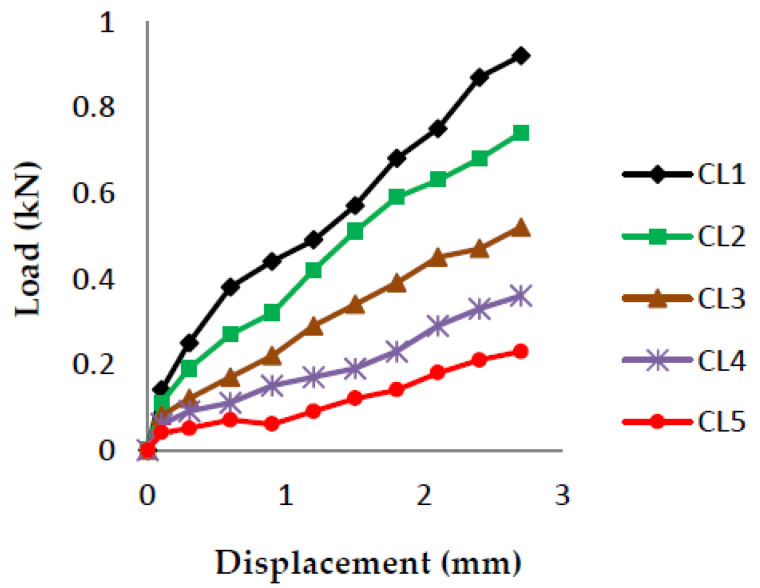
Load vs. Displacement for 70% resin content with banana as filler.

**Figure 14:**
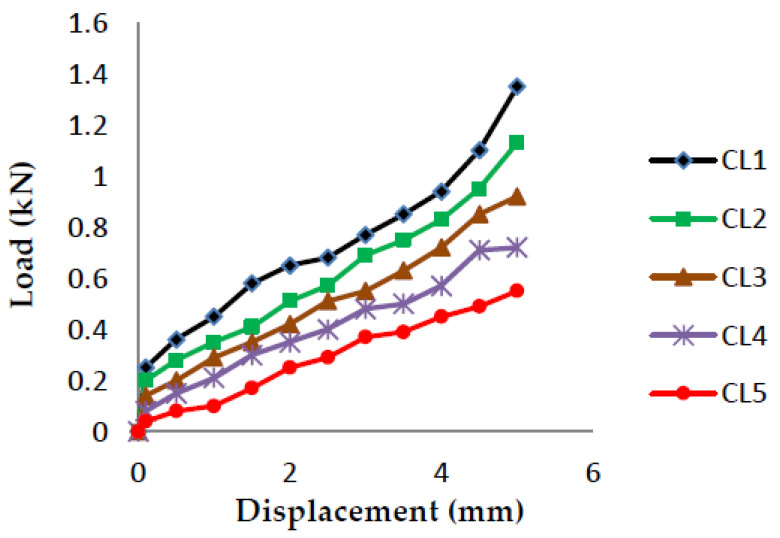
Load vs. Displacement for 70% resin content with coconut shell powder as filler.

**Figure 15:**
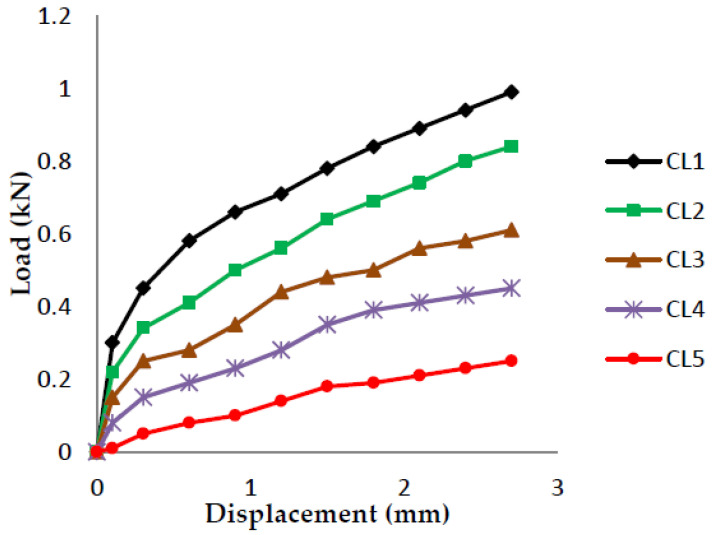
Load vs. Displacement for 70% resin content with sawdust as filler.

The load versus displacement graphs for various crack lengths of 9 mm, 9.5 mm, 10 mm, 10.5 mm, and 11 mm are plotted. The graphs are plotted for various compositions containing different filler materials, such as banana fiber, coconut shell powder, and sawdust, as shown in Figure 7, Figure 8, Figure 9, Figure 10, Figure 11, Figure 12, Figure 13, Figure 14, Figure 15. As the crack length increases, the load applied to attain failure starts decreasing, so the load-carrying capacity of the material decreases. The load required for the 9 mm crack length is higher when compared to the 11 mm crack length.

### 4.2. Compliance v/s Crack Length

Compliance is the tolerance exhibited by a material that undergoes deformation. It is the inverse of stiffness and has the property of flexibility and the ability to distort easily. The stiffness of an object is its resistance to the deformation to the response force applied, the property of being inflexible and difficult to deform.

**Figure 16:**
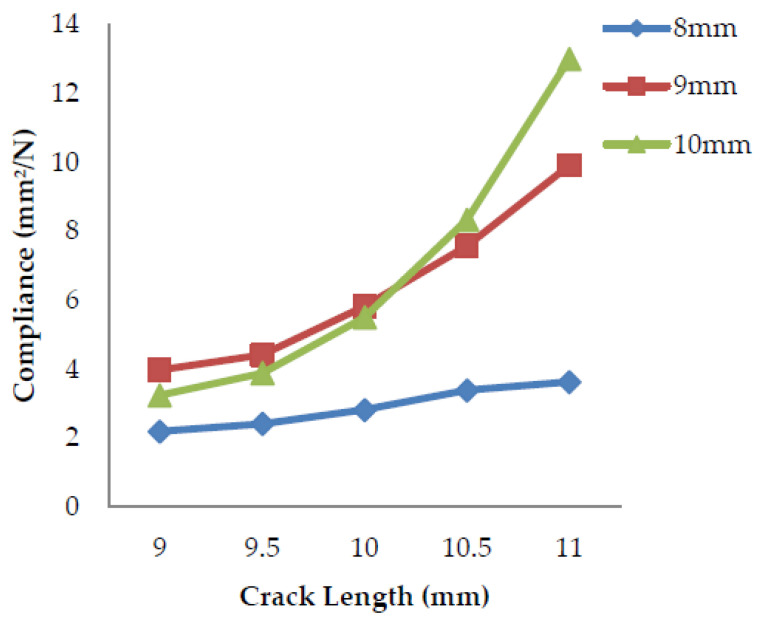
Compliance vs. crack length for banana fiber filler.

**Figure 17:**
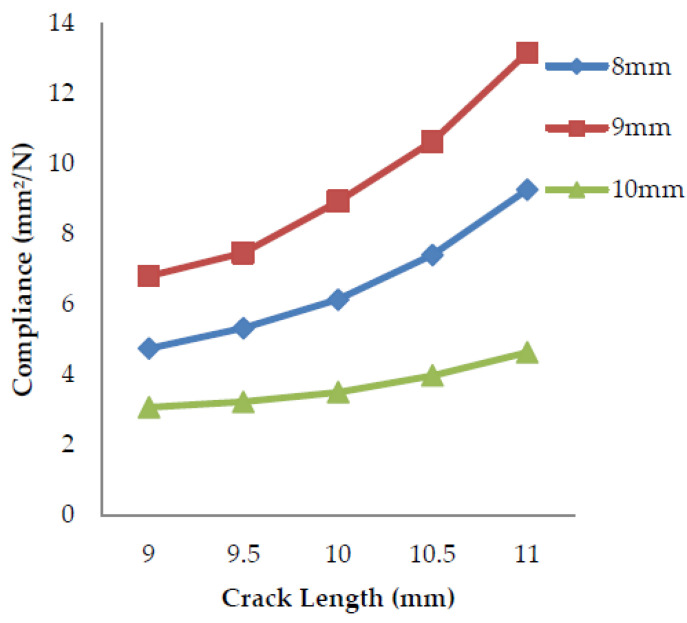
Compliance vs. crack length for coconut shell powder filler.

**Figure 18:**
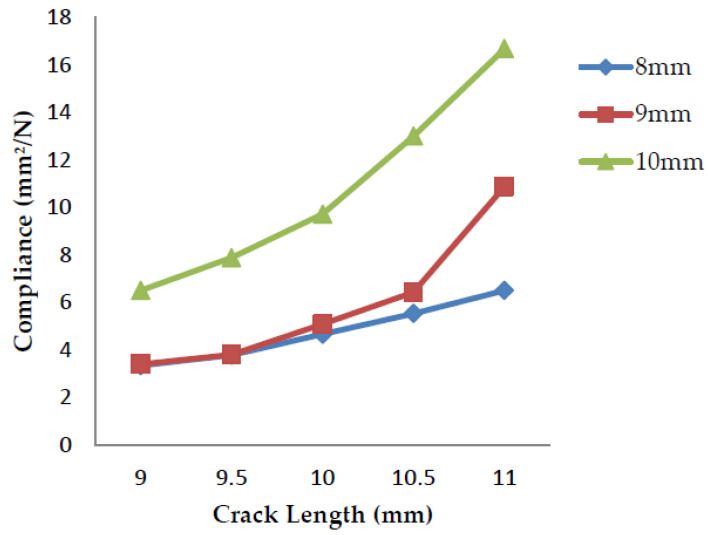
Compliance vs. crack length for sawdust filler.

The compliance of a specimen is determined over a range of crack lengths (9–11 mm). The slope of the load vs. displacement curves of each crack length gives the value of stiffness. The inverse of the stiffness value calculates the compliance. From Figure 16, Figure 17, and Figure 18 above, it can be observed that as the crack length increases, the load applied to the material goes on decreasing and in turn, the stiffness of the material decreases, which leads to an increase in the compliance as it is the inverse of the stiffness. Thus, the compliance is lower for the specimens having crack lengths of 9 mm and higher for the 11 mm rack length.

### 4.3. Fracture Toughness v/s Thickness Graph

The relationship between the thickness of the specimen and fracture toughness is obtained from the following graphs.

**Figure 19:**
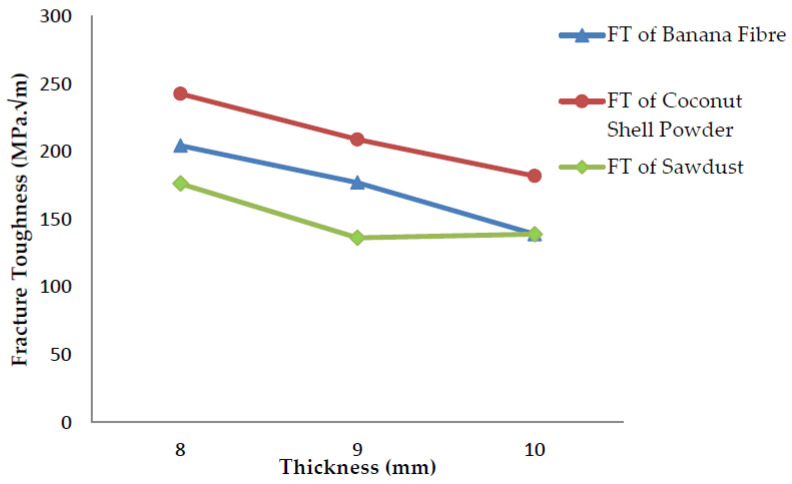
Fracture toughness vs. thickness for three different fillers.

When the thickness increases, the stress–strain region in the crack begins to change from plain stress conditions to plain strain conditions, which means the crack tip is in the condition of tension in all three directions, and the plastic zone will be limited to a smaller region. Therefore, the fracture toughness decreases with an increase in the thickness, as shown in Figure 19. 

## 5. Significance and Outcome

The current study will lead to the development of alternative materials suitable for use in light-duty automotive applications or as packaging material for consumer products. As greenhouse emissions are a major focus in today’s world, this research tries to reduce the carbon footprint by using organic hemp fiber as the natural fiber material along with coconut shell powder, sawdust, and banana fiber as fillers. 

## 6. Conclusions

Natural fiber composites were prepared using hemp fiber and three different fillers, namely, banana fiber, coconut shell powder, and sawdust, with thicknesses of 8, 9, and 10 mm, at 60%, 65%, and 70% of resin contents. These composites were subjected to tensile and fracture toughness tests according to ASTM standards. The effects of the factors mentioned above on the fracture toughness were evaluated using variance (ANOVA) analysis and response surface methodology. From this experimental study, the following conclusions can be drawn:

- The use of natural fibers is beneficial, as carbon fibers are harmful to the environment.
- The maximum fracture toughness value is obtained for the composite of 8 mm thickness, containing 35% of hemp fiber with coconut shell powder as the filler.
- An L9 orthogonal array is used as it is more suitable for analyzing the effects of three different factors at three different levels.
- The Taguchi technique of the L9 orthogonal array helps in the determination of the optimum levels of the tests conducted.

## Figures and Tables

**Table 1:** Details of the material suppliers.

Materials	Suppliers
Hemp fiber	D K Enterprises Hubli, 580031, Karnataka, India.
Epoxy Resin Lapox L12 Epoxy Hardener Lapoxy K6	Yuje Enterprises Bangalore, 560003, Karnataka, India.
Coconut Shell Powder,	Kasturi Coconut Processing, Bangalore, 526160, Karnataka, India.
Banana Fiber	Sindhu Enterprises, Maruti Nagar, Bangalore, 560068, Karnataka, India.
Saw Dust Powder	Zayd Solutions Bangalore, 560084, Karnataka, India.

**Table 2:** Properties of epoxy [23].

Properties	Value
Tensile Strength	68–80 Mpa
Specific Strength	56.6–66.6 kPa m^3^/kg
Elastic Modulus	2.9–3.2 MPa
Deformation	5%–7%
Density	1.2 g/cc

**Table 3:** Process parameters and levels.

Parameters	Level 1	Level 2	Level 3
Hemp fiber content (%)	35	30	25
Banana fiber content (%)	5	5	5
Coconut shell powder (%)	5	5	5
Sawdust (%)	5	5	5
Epoxy resin (%)	60	65	70
Thickness (mm)	8	9	10

**Table 4:** L9 Orthogonal array.

Experimental Trial	Parameter 1	Parameter 2	Parameter 3
1	1	1	1
2	1	2	2
3	1	3	3
4	2	1	2
5	2	2	3
6	2	3	1
7	3	1	3
8	3	2	1
9	3	3	2

**Table 5:** Three point bending test results.

Sl. No.	Resin (%)	Hemp Fiber (%)	Thickness (mm)	Fracture Toughness (MPa.√m)
1	60	35(B) *	8	204.37
2	60	35(C) *	9	208.91
3	60	35(S) *	10	138.97
4	65	30(B) *	9	177.12
5	65	30(C) *	10	181.89
6	65	30(S) *	8	176.27
7	70	25(B) *	10	138.97
8	70	25(C) *	8	242.69
9	70	25(S) *	9	136.25

* (B)—Banana fiber as a filler material; * (C)—Coconut shell powder as a filler material; * (S)—Sawdust as a filler material.

**Table 6:** Analysis of variance for S/N ratio.

Source	Degrees of Freedom	Sum of Squares	Mean Sum of Squares	F Ratio	*p* Value	% Contribution
Thickness in mm	2	10.6043	5.3021	24.67	0.039	42.21
Resin content in %	2	0.9393	0.4697	2.19	0.314	3.73
Fiber content in %	2	13.1487	6.5774	30.59	0.032	52.33
Error	2	0.4298	0.2149			1.71
Total	8	25.1221				

**Table 7:** Response table for signal to noise ratios—larger is better.

Level	Resin Content in %	Fiber Content in %	Thickness in mm
1	45.16	44.68	46.28
2	45.03	46.43	44.68
3	44.42	43.49	43.64
Delta	0.74	2.94	2.64
Rank	3	1	2

## Data Availability

Not applicable.
